# Psychometric Qualities of the Educational Identity Processes Scale (EIPS)

**DOI:** 10.3389/fpsyg.2022.861220

**Published:** 2022-04-19

**Authors:** Annabelle H. T. Christiaens, Stefanie A. Nelemans, Elisabeth L. de Moor, Rasa Erentaitė, Rimantas Vosylis, Susan Branje

**Affiliations:** ^1^Department of Youth and Family, Faculty of Social and Behavioural Sciences, Utrecht University, Utrecht, Netherlands; ^2^Faculty of Social Sciences, Arts, and Humanities, Kaunas University of Technology, Kaunas, Lithuania; ^3^Institute of Psychology, Mykolas Romeris University, Vilnius, Lithuania

**Keywords:** educational identity development, assessment, secondary school transition, context dependencies, adolescence

## Abstract

In the educational domain, the development of identity becomes especially salient during school transition phases. To assess the specific identity processes that match the adolescents' experiences before and after the school transition, the Educational Identity Processes Scale (EIPS) was developed. The present study aimed to test the psychometric qualities of the EIPS by examining its factor structure, the internal and convergent validity of the identity dimensions, and whether the questionnaire was measurement invariant over time. The pre-transition version was tested in a Dutch sample (*N* = 242 early adolescents) and the post-transition version was tested in a Lithuanian sample (*N* = 1,268 mid-adolescents). Findings indicated good psychometric qualities for both the pre- and post-transition versions of the EIPS. Additionally, context dependencies were observed, as distance to the transition influenced the meaning of specific identity processes and determined whether specific processes could be considered as part of normative development.

## Introduction

Identity development concerns finding a clear and coherent sense of self (Erikson, 1963). The development of one's identity becomes a critical task during adolescence, as biological, cognitive, and social changes stimulate young people to reflect on questions such as “Who am I?” and “Who do I wish to become?” (Marcia, 1993). The educational domain is one of the most important identity domains during adolescence, as it is the place where youth spend a lot of their time and are encouraged to think about their future by making concrete choices. Educational identity reflects finding out who one is and which direction one wants to go regarding one's school, curriculum, academic track, and vocational profile (Grotevant, 1987). Although adolescents might reflect on these issues during various periods, these questions become particularly salient during school transition phases, such as the transition from primary to secondary education and from lower to upper secondary education. The present study examined the psychometric properties of the Educational Identity Processes Scale (EIPS), developed to assess the specific identity processes before and after the school transition in a pre- and post-transition context.

### Processes of Educational Identity Development

According to dual cycle models of identity, which are based on the identity status paradigm (Marcia, 1966), adolescents form their identity through processes of exploration and commitment (Crocetti et al., 2008; Luyckx et al., 2008). Adolescents start to develop their educational identity through a formative cycle, in which they use exploration in-breadth to search for and explore different identity alternatives, and select and commit to one of these alternatives as a direction in life. Thus, in the context of an approaching school transition, adolescents might compare different schools, reflect on which school would fit them best, and select a school based on their options and preferences. Once adolescents have made a commitment to an identity choice, a maintenance cycle starts in which adolescents explore their identity choice in depth by actively reflecting on their performance and preferences, and identify with their commitment by integrating it with their interests, beliefs, future goals, and values. That is, once adolescents have started at their new school, they will reflect on whether the school environment and curriculum matches their interests and abilities, and based on this reflection they will strengthen or weaken their identification with the school.

When the selected school is deemed unsatisfactory, adolescents can reconsider their commitment. This means that after the transition, adolescents can start to look for different schools to find a better alternative and discuss their doubts about their current selected school with others, thus returning adolescents to the formative cycle (Crocetti et al., 2008; Luyckx et al., 2008). Therefore, reconsideration and exploration in-breadth are comparable identity processes that tap into the same behavior of searching for possible new commitments, but theoretically the identity processes differ by either the presence or absence of current commitments, respectively (Crocetti et al., 2008). Finally, adolescents may experience self-doubt about their ability to find the best-fitting commitment (Luyckx et al., 2008; Porfeli et al., 2011). Surrounding the school transition, adolescents can doubt whether they will find the right school and worry if they actually like their selected school. The process self-doubt is related to exploration in-breadth and reconsideration, but differs as these latter two options capture a willingness to search for a new school while self-doubt captures a passive ruminative response through which adolescents can get stuck in a state of uncertainty about their education (Porfeli et al., 2011).

### Development of the Educational Identity Processes Scale (EIPS)

Existing identity measures typically assess identity processes in an abstract and decontextualized manner, by either focusing on one's sense of commitment and exploration in general [e.g., Dimensions of Identity Development Scale (DIDS), Luyckx et al., 2008] or in specific domains [e.g., Utrecht-Management of Identity Commitments Scale (U-MICS), Crocetti et al., 2008]. As the items of these measures do not take into account the specific normative context, they risk being non-relevant for adolescents at the time of filling out the questionnaire. Particularly in the domain of educational identity, which is considered a closed identity domain where change in development is constrained by normative educational transitions (Meeus et al., 1999). That is, the question of whether one is considering going to another school is not relevant for most adolescents during periods in which they do not have any need or options to change schools. In addition to being non-relevant, a too general assessment of identity processes in the educational domain also risks not capturing distinctive characteristics of specific identity processes. During normative school transitions adolescents are required to make informed decisions about their future and will encounter several concrete experiences in their newly selected school environment that can be used to evaluate their commitments (Kalakoski and Nurmi, 1998). The role of timing and context is thus important when studying identity (Bosma and Kunnen, 2001), and transitional periods might pose a challenge to adolescents' identity (Branje et al., 2021; Christiaens et al., 2021) as well as the current measurement thereof.

To facilitate and encourage context-specific identity research in the educational domain that is connected to the real-life challenges of adolescents, we developed a new questionnaire with two versions to capture the specific identity processes that occur before and after a school transition: the Educational Identity Processes Scale (EIPS). All authors of the present study and several experts in the field of identity development were involved in the development and translation of the questionnaire (translated and backtranslated from Dutch to English and from English to Lithuanian). The EIPS builds on theoretical identity models (Marcia, 1966; Grotevant, 1987) and is inspired by previous identity measures [e.g., DIDS, Luyckx et al., 2008; U-MICS, Crocetti et al., 2008; Vocational Identity Status Assessment (VISA), Porfeli et al., 2011; see Supplementary Tables S2, S4 for an overview of the set-up of the EIPS], as well as suggestions for improvements from Waterman (2015). Specifically, the pre-transition questionnaire applies to the period just before a normative educational transition and captures the formative cycle of identity development with future-oriented processes of commitment making and exploration in-breadth that focus on the goal of searching for and comparing a range of viable educational options (e.g., “I think actively about which school I like most”) to select a new school or type of education (e.g., “I know which school I want to attend”). Additionally, adolescents can reflect on whether these viable educational options are connected to their own interests and preferences with the processes exploration in-depth (e.g., “I often reflect on which courses I am good at and less good at”) and identification with commitment (e.g., “The school I want to attend really fits me”). Once adolescents have made the transition, the processes of commitment making and exploration in-breadth become less salient and adolescents evaluate their present commitments through concrete hands-on experiences in this newly selected educational environment. Therefore, the post-transition questionnaire captures the identity maintenance cycle through context-relevant processes of exploration in-depth (e.g., “I often reflect on which courses I am good at and less good at”), identification with commitments (e.g., “My school really fits me”), and identity reconsideration (e.g., “I often think that a different school would fit me better”). We also included a more general sense of self-doubt both pre- and post-transition (e.g., “I worry about which school I really like”/“I worry if I really like my school”).

### Present Study

The present study aimed to test the psychometric qualities of the EIPS through three primary objectives, which were pre-registered on OSF (https://osf.io/5d392): 1) to test the factor structure of the questionnaire, 2) to test the internal and convergent validity of the identity dimensions, and 3) to test whether the questionnaire is measurement invariant over time. These three objectives were tested separately in two independent longitudinal samples. Study 1 tested the psychometrics of the pre-transition version and Study 2 tested the psychometrics of the post-transition version of the questionnaire.

#### Objective 1: Factor Structure

The first objective was to assess the factor structure and reliability of the pre- and post-transition EIPS. As the dual-cycle models suggest different identity processes to play a role in the identity formation and identity maintenance cycle, we propose that the educational context determines which processes are most likely to occur. Therefore, in line with the development of the EIPS items in relation to the educational context, the number of factors was expected to differ between the pre- and post-transition version of the scale. Specifically, we expected that pre-transition educational identity was captured by a five-factor solution through the dimensions exploration in-breadth, exploration in-depth, commitment making, identification with commitment, and self-doubt. We further expected that post-transition educational identity was captured by a four-factor solution, including the dimensions exploration in-depth, identification with commitment, reconsideration, and self-doubt. We expected good reliability for all EIPS subscales.

#### Objective 2: Validity

The second objective was to test the internal and convergent validity of the EIPS by assessing whether the EIPS dimensions were meaningfully related to each other (i.e., internal validity) and with other constructs they should be related to (i.e., convergent validity). In line with the dual-cycle models of identity, multiple studies have shown positive associations between the dimensions commitment making and identification with commitment, and between exploration in-depth and exploration in-breadth/reconsideration (e.g., Crocetti et al., 2008; Luyckx et al., 2008; Porfeli et al., 2011). Furthermore, research has consistently shown a negative association of self-doubt with commitment making, identification with commitment, and exploration in-depth, as well as a positive association between self-doubt and exploration in-breadth/reconsideration. In the present study, given that the pre- and post-transition version of the EIPS are based on the structure of existing identity measures and dual-cycles models of identity (Crocetti et al., 2008; Luyckx et al., 2008; Porfeli et al., 2011), and that the educational identity processes are centered around the school transition, we expected similar associations as observed by research on existing identity measures.

For convergent validity, in general, commitment processes are an adaptive part of identity development that have been associated with more positive outcomes (for a review see Branje et al., 2021). In contrast, self-doubt generally hinders the identity process and has been related to more negative outcomes (e.g., Beyers and Luyckx, 2016). Finally, while exploration in-depth and in-breadth are important processes that help adolescents in making well thought-through commitments, they also appear to have a less adaptive side through associations with elevated anxiety and distress (e.g., Luyckx et al., 2008). Therefore, we expected commitment (i.e. commitment making and identification with commitment) to be positively associated with adaptive outcomes, such as self-concept clarity (Schwartz et al., 2011), wellbeing (e.g., Crocetti et al., 2008; Luyckx et al., 2008), and academic motivation (Kaplan and Flum, 2009), while we expected negative associations of self-doubt and reconsideration with these adaptive outcomes. Additionally, based on previous research on the associations of different social-cognitive styles to handle identity formation with identity processes (Bosch and Card, 2012; Crocetti et al., 2013; Monacis et al., 2016), we expected a positive association of an informational identity style with identification with commitment and exploration in-depth. We expected a negative association of a diffuse-avoidant identity style with identification with commitment, but a positive association with reconsideration and self-doubt. Moreover, we expected higher levels of reconsideration and self-doubt to be associated with more negative outcomes, such as anxiety and depression (only measured pre-transition; e.g., Crocetti et al., 2008; Luyckx et al., 2008) and adolescents' perceived parental doubt about their education (Chatterjee and Sinha, 2013), while we expected higher levels of commitment to be associated with less anxiety, depression and parental doubt. Finally, as exploration has been linked to adaptive and maladaptive outcomes through its reflective and ruminative side (e.g., Luyckx et al., 2008), we explored the association of the dimensions exploration in-breadth and exploration in-depth with constructs of the self, wellbeing, parental reflected doubt, and academic motivation, with and without controlling for self-doubt (i.e., thereby controlling for the ruminative aspect).

Finally, we further explored the validity of the EIPS by examining the Average Variance Extracted (AVE) of each identity dimension (i.e., convergent validity) and comparing the AVE values with Average Shared Variance (ASV) of the other identity constructs and covariates (i.e., discriminant validity; Hair et al., 2009; Cheung and Wang, 2017).

#### Objective 3: Measurement Invariance

For both versions of the EIPS, we expected measurement invariance over a period of 6 months, in the period closely before or after the school transition (Mastrotheodoros and Motti-Stefanidi, 2017). Specifically, we expected that over time the scale had a similar factor structure (i.e., configural invariance), the items were equally important to the measurement of the identity subscales (i.e., metric invariance), the items had equal levels and scaling (i.e., scalar invariance), and that differences between measurement waves in the items are due only to differences in the common factors (i.e., full uniqueness invariance). Additionally, in Study 2 we explored measurement invariance across gender to examine if the EIPS assesses educational identity identically in males and females, since research has highlighted gender differences in adolescent identity development (e.g., Verschueren et al., 2017).

## Study 1: Pre-Transition EIPS

### Methods

#### Participants

The sample of Study 1 consisted of 242 adolescents (50.0% female) from the Netherlands with an average age of 11.56 years old (*SD* = 0.44, range = 10–12 years old), who were all in the final grade of primary education. Most respondents identified as Dutch (96.7%) and rated their socio-economic status compared to other people in the Netherlands as medium to high with an average score of 7.7 on a scale from 1 to 10 (*SD* = 1.11, range = 3–10; Goodman et al., 2001). For the second measurement wave, 194 respondents agreed to participate (80.2% retention rate), but 3 participants were excluded due to poor response quality resulting in a study sample in W2 of 191 adolescents. Participants included in both waves did not differ from participants who dropped out or were excluded based on gender, $\chi_{\left(1\right)}^{2}$ = 3.01, *p* = 0.083, or socioeconomic status, *t*_(240)_ = 0.29, *p* = 0.773, but excluded adolescents were slightly older compared to included adolescents, *t*_(239)_ = −2.41, *p* = 0.017. Incidental missing data were random across measurement waves as indicated by a non-significant Little's MCAR test, $\chi_{\left(200\right)}^{2}$ = 208.50, *p* = 0.326 (Kline, 2016).

#### Procedure

Data from the Study 1 were part of the INTRANSITION project, a longitudinal research project with four measurement waves across 2 years focused around the school transition. Adolescents in the final year of primary school were recruited through primary schools across the Netherlands. The 84 schools that agreed to cooperate, sent out information and consent forms to the parents and allowed promotion of the project on their communication platforms and through small promotional talks. Before the start of the study, both adolescents and one of their parents provided active informed consent. The first assessment took place between October 2019 and February 2020 and the second assessment started 6 months later between May and July 2020, toward the end of the final grade of primary school. Therefore, these first two waves of the project were used in Study 1 to assess the adolescent's pre-transition experiences. Questionnaires were completed individually online. Each questionnaire lasted around 60–90 min and adolescents received a monetary reward of €10 for each assessment. The INTRANSITION project was approved by the local Ethics board of Utrecht University (protocol no. FETC18-135, FETC20-157).

#### Measures

##### Educational Identity

At the end of primary school, adolescents in the Netherlands need to select a new school and choose an educational trajectory that fits with their capabilities, interests, and future goals. Pre-transition educational identity was assessed through the EIPS scale (see Supplementary Table S2 for the complete pre-transition version of the scale), which consisted of the subscales “exploration in-breadth” (5 items), “exploration in-depth” (5 items), “commitment making” (3 items), “identification with commitments” (4 items), and “self-doubt” (5 items). The items were assessed on a scale of 1 (*completely disagree*) to 5 (*completely agree*).

##### Self-concept Clarity

The degree to which adolescents have a clear and consistent image of their self was assessed with the Self-concept Clarity scale (Campbell et al., 1996). The measure consisted of 12 items (e.g., “My beliefs about myself often conflict with one another;” α = 0.84) that were rated on a scale from 1 (*strongly disagree*) to 5 (*strongly agree*). The answers were recoded, such that a higher score indicated higher self-concept clarity. Validity has been demonstrated by Crocetti et al. (2008).

##### Wellbeing

As a positive marker of wellbeing, life satisfaction was assessed with the Cantril Ladder (Cantril, 1965). Adolescents rated the question “How do you feel in general?” on a scale from 1 to 10 to indicate their degree of general life satisfaction, with 10 indicating the most life satisfaction. Validity was demonstrated (Levin and Currie, 2014) and in the present study test-retest reliability was low but significant between measurement waves (*r* = 0.38, *p* < 0.001). Additionally, as a negative marker of wellbeing, symptoms of “Generalized Anxiety Disorder” (GAD) and “Major Depressive Disorder” (MDD) were assessed with the Revised Child Anxiety and Depression scale (Chorpita et al., 2005). The subscale GAD consisted of 6 items (e.g., “I worry about things;” α = 0.82) and the subscale MDD consisted of 10 items (e.g., “I feel sad or empty;” α = 0.80). The items were rated on a scale from 1 (*never*) to 4 (*always*). Validity of the scale has been demonstrated by Chorpita et al. (2005).

##### Parental Reflected Doubt

Adolescents rated the degree they perceived parental doubt about their education on a scale of 1 (*strongly disagree*) to 5 (*strongly agree*). The scale consisted of 5 items (e.g., “My parents doubt whether my school really fits me;” α = 0.81) and was developed for the INTRANSITION project (see Supplementary Table S6 for the full scale).

##### Academic Motivation

Academic motivation was assessed with the Academic Self-Regulation scale (Ryan and Connell, 1989). Adolescents rated the degree to which they were academically motivated for its own sake (e.g., out of interest or excitement) with the subscale “intrinsic motivation.” The subscale consisted of 7 items (e.g., “When I do my homework, I do this because I like doing my homework;” α = 0.90) that were rated on a scale from 1 (*completely disagree*) to 5 (*completely agree*). Validity has been demonstrated by Vansteenkiste et al. (2009).

#### Analyses

To examine the facture structure of the pre-transition questionnaire (Objective 1), we conducted a Confirmatory Factor Analysis (CFA) on the first measurement wave using the robust maximum likelihood estimator (i.e., MLR estimator) in Mplus Version 8.3 (Muthén and Muthén, 1998–2017). The conceptual model consisted of the following five latent variables: “exploration in-breadth” (5 items), “exploration in-depth” (5 items), “commitment making” (3 items), “identification with commitment” (4 items), and “self-doubt” (5 items). Items were only allowed to load on a single factor. If the conceptual model did not show acceptable fit to the data, modification indices were consulted and used for model improvement if these were conceptually supported. First, we examined possible item misfit, followed by possible correlations between error terms. The final CFA model of the first measurement was then also examined in the second measurement wave to inspect the longitudinal stability of the model. Lastly, for the final CFA model we inspected the reliability with Cronbach's coefficient alpha (Cronbach, 1951) and Raykov's rho (composite reliability; Raykov, 2001).

For Objective 2, we computed means scores when at least 70% of the items within the (sub)scales were completed to examine the internal and convergent validity of the pre-transition questionnaire in W1. When there were missing data on the level of the scale score, missingness was imputed with the expectation-minimization algorithm (EM) using the remaining scores as predictors of those values. Associations between the identity dimensions and possible related constructs at W1 were examined through bivariate Pearson correlation analyses in SPSS Version 25. Additionally, we examined partial correlations of the dimensions exploration in-breadth and exploration in-depth with possible related constructs controlling for self-doubt. To further explore the convergent validity of the questionnaire, we examined the Average Variance Extracted (AVE) that captures the proportion of variance in the items in a subscale explained by the factor as compared to non-attributable factors (i.e., error; Cheung and Wang, 2017). AVE values of 0.50 or higher would indicate good convergence between items of the same scale as at least half of the variance in the items is explained by the latent factor and not by error (Hair et al., 2009). For discriminant validity, we examined whether AVE values were higher than the Average Shared Variance (ASV) values of the other identity constructs and covariates (Hair et al., 2009).

We examined longitudinal measurement invariance of the EIPS (Objective 3) across two measurement waves 6 months apart for each identity dimension separately to reduce model complexity (Vandenberg and Lance, 2000). To account for interdependencies between the assessments, residual covariances between the same items over time were included in all models as recommended by Vandenberg and Lance (2000). To test for configural invariance, we specified a CFA model based on the final selected model for Objective 1 by including scale-specific adjustments. Next, we specified three consecutive and nested CFA models with increasing equality constraints to test for metric (i.e., equality constraints to the factor loadings), scalar (i.e., equality constraints to the item intercepts), and full uniqueness invariance (i.e., equality constraints to the error variances across assessments; Van Der Schoot et al., 2015). If full invariance was not achieved in one of the models, partial invariance was tested by relaxing equality constraints based on modification indices and theoretical meaning.

Acceptable fit to the data was indicated by a CFI ≥ 0.90, RMSEA ≤ 0.10, and SRMR ≤ 0.10 (Browne and Cudeck, 1993; Hu and Bentler, 1999). At least two out of three criteria needed to be met to indicate acceptable fit for model selection. Additionally, measurement invariance was assumed to hold when fit of the model with increasing constraints did not significantly worsen, according to the following criteria: ΔCFI ≤ −0.005 together with ΔRMSEA ≤ 0.010 or ΔSRMR ≤ 0.025 for metric invariance, but ΔSRMR ≤ 0.005 for scalar and full uniqueness invariance (Chen, 2007).

### Results

#### Factor Structure

**Figure 1:**
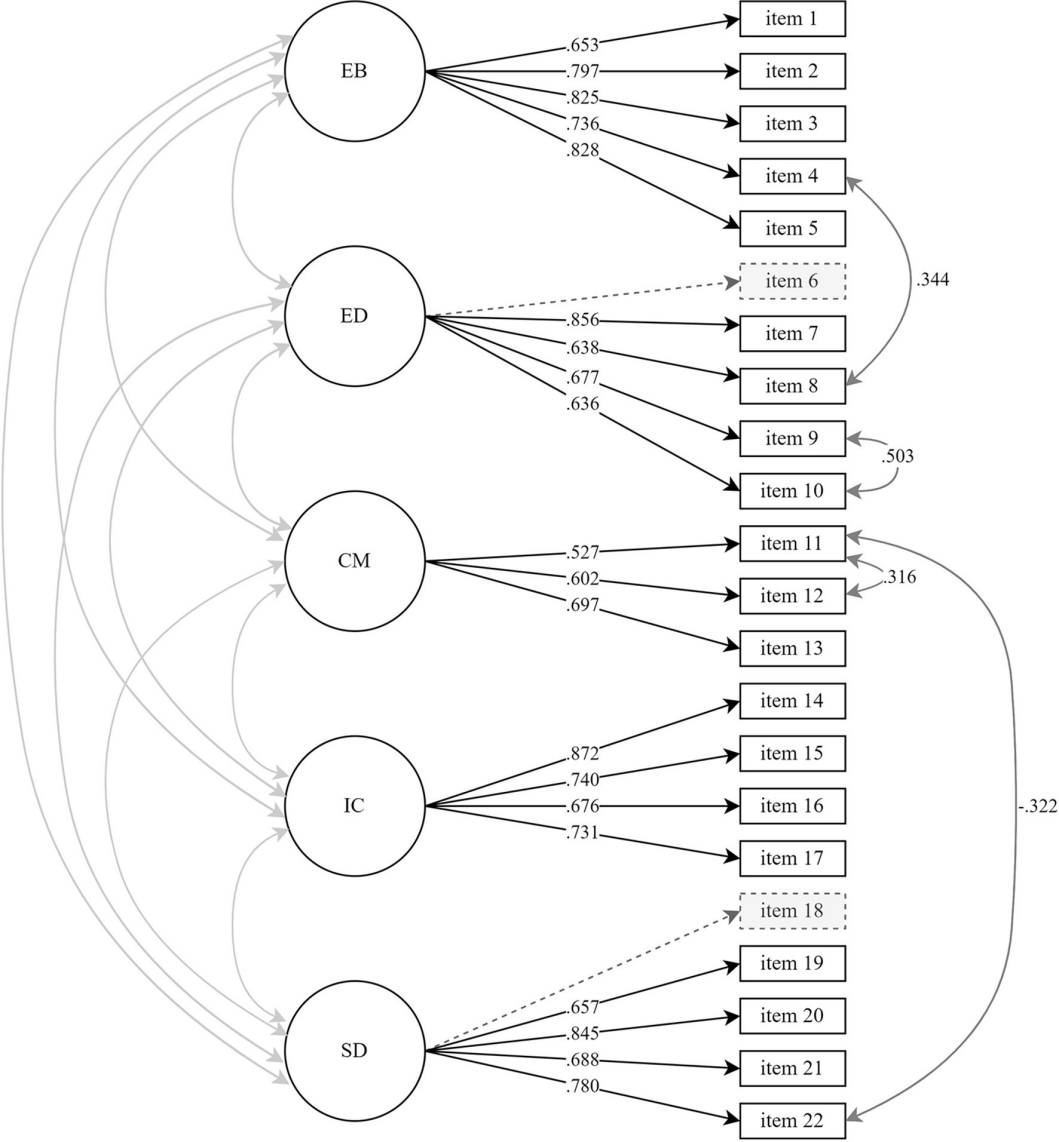
Final 5-factor model pre-transition questionnaire (W1) with standardized estimates. Changes from the conceptual model are marked with dotted markings, indicating the removal of items 6 and 18, and with dark grey arrows, indicating correlation between error terms. EB, exploration in-breadth; ED, exploration in-depth; CM, commitment making; IC, identification with commitments; SD, self-doubt. *n* = 230.

Two out of three model fit criteria indicated that the conceptual model had an acceptable fit to the data (CFI = 0.829, RMSEA = 0.087, SRMR = 0.085). However, as the CFI index was substantially below the established threshold, we decided to deviate from the pre-registration and more closely inspect the model to identify sources of a misfit. After slight adjustments, including removal of two items and adding error-correlation terms between a few items (Figure 1 and Supplementary Table S3), the model showed acceptable fit according to all three criteria, CFI = 0.907, RMSEA = 0.066, SRMR = 0.072. Reliability was overall sufficient, as indicated by α and ρ values ≥0.70, for the final estimated model (Table 1 and Supplementary Table S1), although composite reliability of commitment making in the final 5-factor model was ρ = 0.64.

**Table 1:** Descriptive statistics and internal validity of the pre-transition EIPS.

	**Descriptives**					**Zero-order correlations**			
	**α**	***M***	***SD***	**AVE**	**ASV**	**Exploration in-breadth**	**Exploration in-depth**	**Commitment making**	**Identification with commitment**
Exploration in-breadth	0.88	3.60	0.89	0.59	0.10	–			
Exploration in-depth	0.84	3.35	0.85	0.50	0.11	0.527^***^	–		
Commitment making	0.70	3.55	0.91	0.38	0.14	0.032	0.180^**^	–	
Identification with commitment	0.84	3.99	0.70	0.57	0.17	0.318^***^	0.331^***^	0.562^***^	–
Self-doubt	0.83	1.95	0.80	0.56	0.09	−0.065	0.039	−0.438^***^	−0.410^***^

*N = 242*.

*p < 0.05;

** *p < 0.01;*

*** *p < 0.001*.

*α, Cronbach's alpha of the final 5-factor model at W1*.

*For the complete assessment of the reliability of the pre-transition version of the EIPS (see Supplementary Table S1). AVE, average variance extracted; ASV, average shared variance*.

Next, we examined the longitudinal stability of the model in W2 by first testing the initial conceptual model and subsequently inspecting whether adjustments from W1 also applied to W2. Results showed acceptable fit of the initial conceptual model according to all three criteria in W2, CFI = 0.952, RMSEA = 0.044, SRMR = 0.054. Model fit improved when applying the suggested W1 adjustments, CFI = 0.980, RMSEA = 0.029, SRMR = 0.046, although the reliability of commitment making was again low, α = 0.49 and ρ = 0.53 (Supplementary Table S1). Therefore, the results generally show that after a few adjustments, the 5-factor structure of the pre-transition version of the EIPS fit well to both waves of the data, although the commitment making scale showed some signs of misfit and unreliability.

#### Validity

With few exceptions, all expected associations between identity dimensions were in line with our prediction, although effect sizes ranged from weak to strong, providing evidence of internal validity. However, the association of self-doubt with the exploration dimensions was not significant (Table 1). In addition, we observed a significant positive association of exploration in-depth with both commitment dimensions and a positive association between exploration in-breadth and identification with commitment.

With few exceptions, all correlations between the identity dimensions and the covariates were in the expected direction, although effect sizes ranged from weak to strong. More positive outcomes (i.e., more self-concept clarity, life satisfaction and academic motivation, and less depressive symptoms and parental reflected doubt) were associated with more identification with commitment, while negative outcomes (i.e., more parental doubt, depressive, and anxiety symptoms, and less self-concept clarity, life satisfaction and academic motivation; Table 2) were associated with more self-doubt. Only five expected correlations were not significant: the negative associations between identification with commitment and anxiety and between self-doubt and academic motivation, and the positive associations of commitment making with self-concept clarity, wellbeing, and academic motivation. Exploration of the convergent validity of the exploration dimensions indicated a significant positive association of both exploration dimensions with self-concept clarity and academic motivation, with and without controlling for self-doubt (Table 2). Therefore, the findings provided substantial evidence of the internal and convergent validity of the pre-transition EIPS subscales.

**Table 2:** Zero-order and partial correlation testing the convergent validity of the pre-transition EIPS.

	**ASV**	**Self-concept clarity**	**Life satisfaction**	**Anxiety symptoms**	**Depressive symptoms**	**Reflected doubt**	**Academic motivation**
**Zero-order correlations**							
Exploration in-breadth	0.04	0.323^***^	0.048	−0.124	−0.106	−0.114	0.273^***^
Exploration in-depth	0.03	0.164^*^	0.097	−0.055	−0.06	−0.01	0.358^***^
Commitment making	0.01	0.025	−0.002	0.004	−0.006	−0.216^**^	0.089
Identification with commitment	0.05	0.218^**^	0.142^*^	−0.123	−0.245^***^	−0.333^***^	0.218^**^
Self-doubt	0.08	−0.319^***^	−0.181^**^	0.199^**^	0.292^***^	0.461^***^	−0.108
**Partial correlations**							
Exploration in-breadth	0.03	0.319^***^	0.037	−0.114	−0.092	−0.095	0.268^***^
Exploration in-depth	0.03	0.186^**^	0.106	−0.065	−0.075	−0.031	0.364^***^

*N = 242. Partial correlations are controlled for self-doubt. ASV, average shared variance*.

* *p < 0.05;*

** *p < 0.01;*

*** *p < 0.001*.

Finally, exploratory analyses further supported the validity of the pre-transition version of the EIPS. AVE values were generally above 0.50, indicating high convergence between the items within each dimension (Table 1), and AVE values of each identity dimension were higher than the ASV values of other identity dimensions (Table 1) and covariates (Table 2), indicating discriminant validity.

#### Measurement Invariance

The configural longitudinal invariance models had a good fit to the data, however, the inclusion of factor loading constraints decreased model fit. Findings indicated at least (partial) metric invariance for all five subscales of the pre-transition questionnaire (Table 3) after relaxing a few equality constraints on the factor loadings. Specially, items 11 and 13 of commitment making and items 14 and 16 of identification with commitment were relaxed to obtain partial metric invariance for these subscales. Further, (partial) scalar invariance and (partial) full uniqueness invariance were found for exploration in-breadth and exploration in-depth, after relaxing specific equality constraints on the item intercepts and error variances for item 1 of exploration in-breadth. Therefore, the subscales commitment making, identification with commitment, and self-doubt can be used to test associations over time, while the exploration in-breadth and exploration in-depth can also be used to examine mean-level development over time.

**Table 3:** Model fit for the different levels of pre-transition measurement invariance over time.

**Identity process/model**	**CFI**	**ΔCFI**	**RMSEA**	**ΔRMSEA**	**SRMR**	**ΔSRMR**
**Commitment making (3 items)** ^a^						
Model 1: Configural invariance	1.000		0.000		0.021	
Model 2: Metric invariance	0.800	−0.200	0.114	0.114	0.114	0.093
Model 3: Partial metric invariance^b^	1.000	0.000	0.000	0.000	0.045	0.024
Model 4: Scalar invariance	0.106	−0.894	0.228	0.228	0.165	0.120
Model 5: Partial scalar invariance^b^	0.593	−0.407	0.172	0.172	0.091	0.046
**Identification with commitment (4 items)**						
Model 1: Configural invariance	0.959		0.069		0.049	
Model 2: Metric invariance	0.926	−0.033	0.082	0.013	0.195	0.146
Model 3: Partial metric invariance^c^	0.956	−0.003	0.067	−0.002	0.080	0.068
Model 4: Scalar invariance	0.832	−0.124	0.118	0.051	0.158	0.078
Model 5: Partial scalar invariance^d^	0.938	−0.018	0.075	0.008	0.106	0.026
**Exploration in-breadth (5 items)**						
Model 1: Configural invariance	0.986		0.039		0.041	
Model 2: Metric invariance	0.990	0.004	0.031	−0.008	0.051	0.010
Model 3: Scalar invariance	0.955	−0.035	0.060	0.029	0.069	0.018
Model 4: Partial scalar invariance^e^	0.989	−0.001	0.030	−0.001	0.058	0.007
Model 5: Full uniqueness invariance	0.979	−0.010	0.040	0.010	0.089	0.031
Model 6: Partial full uniqueness invariance^f^	0.987	−0.002	0.032	0.002	0.078	0.020
**Exploration in-depth (4 items)**						
Model 1: Configural invariance	1.000		0.000		0.020	
Model 2: Metric invariance	1.000	0.000	0.000	0.000	0.037	0.017
Model 3: Scalar invariance	1.000	0.000	0.000	0.000	0.042	0.005
Model 4: Full uniqueness invariance	1.000	0.000	0.000	0.000	0.049	0.007
**Self-doubt (4 items)**						
Model 1: Configural invariance	0.968		0.063		0.049	
Model 2: Metric invariance	0.973	0.005	0.052	−0.011	0.072	0.023
Model 3: Scalar invariance	0.901	−0.072	0.090	0.038	0.113	0.041
Model 4: Partial scalar invariance^g^	0.932	−0.041	0.077	0.025	0.091	0.019

a *Variance of W2 item 12 was set to 0 as this item had a non-significant negative variance*.

b *Equality constraints of item 11 and item 13 were released for partial metric and scalar invariance, no further suggestions for improvement were possible*.

c *Equality constraints of item 14 and item 16 were released for partial metric invariance*.

d *Equality constraints of item 14 and item 15 were released for partial scalar invariance, no further suggestions for improvement were given*.

e *Equality constraint of item 1 was released for partial scalar invariance*.

f *Equality constraint of item 1 was released for partial full uniqueness invariance*.

g *Equality constraint of item 21 was released for partial scalar invariance, no further suggestions for improvement were given*.

## Study 2: Post-Transition EIPS

### Methods

#### Participants

The sample of Study 2 consisted of 1,268 adolescents (52.1% female) from Lithuania, with an average age of 14.87 years old (*SD* = 0.39, range = 14–16), who recently transitioned to upper secondary education. Most respondents identified as Lithuanian (98.8%), with a heterogeneous representation of socio-economic background: 12.9% received free nutrition at school (comparable to the national average in 2019; National Agency for Education (Lithuania), 2022). Only respondents who participated during the first wave were contacted for the second wave, and 1,204 (94.9%) respondents agreed to do so. Respondents that dropped out of the study were slightly older than the included respondents, *t*_(1,265)_ = 3.80, *p* = < 0.001, and were more likely to be male, $\chi_{\left(1\right)}^{2}$ = 19.88, *p* < 0.001, but did not differ based on socioeconomic status, $\chi_{\left(1\right)}^{2}$ = 2.05, *p* = 0.152. Data were missing at random across measurement waves as indicated by a χ^2^/df ratio < 3, $\chi_{\left(19,210\right)}^{2}$ = 20,646.96, *p* < 0.001, χ^2^/df ratio = 0.93 (Kline, 2016).

#### Procedure

The data were part of the Goals' Lab project, a longitudinal design with four measurement waves across 2 years. The full EIPS questionnaire was provided only in the first and second assessments, during which adolescents were in the first year of upper secondary education. Respondents were recruited through a multi-stage sampling procedure: 14 out of the 60 Lithuanian urban and rural municipalities were selected based on balanced quotas for socio-economic indicators and school achievement levels. Parents and students provided active consent after receiving detailed information on the study. The first assessment consisted of an online questionnaire and took place in October-November 2019 during allocated class hours (45 min). The second assessment took place in April-May 2020 and was administered entirely online from the adolescents' place of residence due to the COVID-19 pandemic. Adolescents were not paid for participation but were informed about the possibility of receiving general feedback on their results after the final assessment of the study. Compliance to the ethical standards was reviewed and approved by the Civil Society and Sustainability research group at the Faculty of Social Sciences, Arts, and Humanities, Kaunas University of Technology (protocol no. V19-1253-12).

#### Measures

##### Educational Identity

In Lithuania, at the start of upper secondary education most adolescents began learning in a new (gymnasium-level) school, although some could remain at their old school (extended gymnasium). Post-transition educational identity was assessed through the EIPS scale (see Supplementary Table S4 for the complete post-transition version of the scale). The post-transition version of the questionnaire consisted of the subscales “exploration in-depth” (5 items), “reconsideration” (6 items), “identification with commitment” (4 items), and “self-doubt” (3 items). The items were assessed on a scale of 1 (*completely disagree*) to 5 (*completely agree*).

##### Identity Style

Adolescents' identity style was assessed during the first measurement wave with the Identity Style Inventory-5 (Berzonsky et al., 2013). The scale measured three social-cognitive strategies for the task of identity formation: “Informational identity style” capturing the strategy of actively seeking out information (9 items; e.g., “Talking to others helps me explore my personal beliefs;” α = 0.88), “normative identity style” capturing the strategy of conforming to others' expectations (9 items; e.g., “I automatically adopt and follow the values I was brought up with;” α = 0.74), and “diffuse-avoidant identity style” capturing the strategy of avoiding identity issues (9 items; e.g., “I am not really thinking about my future now, it is still a long way off;” α = 0.71). The items were assessed on a scale of 1 (*disagree a lot*) to 5 (*agree a lot*). Validity was demonstrated by Berzonsky et al. (2013).

##### Wellbeing

Satisfaction with life was assessed during the second measurement wave with the Satisfaction with Life Scale-Child (Gadermann et al., 2010). The scale consisted of 5 items (e.g., “In most ways my life is close to the way I would want it to be;” α = 0.84) that were rated on a scale of 1 (*disagree a lot*) to 5 (*agree a lot*). Validity was demonstrated by Gadermann et al. (2010).

##### Parental Reflected Doubt

Adolescents rated the degree they perceived parental reflected doubt about their education during the second measurement wave on a scale of 1 (*strongly disagree*) to 5 (*strongly agree*). The scale consisted of 6 items (e.g., “My parents doubt whether my school really fits me;” α = 0.81) and was developed for the INTRANSITION project (see Supplementary Table S6 for the full scale).

##### Academic Motivation

Adolescents rated the degree to which their academic achievement goal was focused on attaining intrapersonal competence during the second measurement wave on the “mastery-approach” subscale of the Achievement Goal Orientation scale (Elliot and Murayama, 2008). The subscale consisted of 3 items (e.g., “My goal is to learn as much as possible;” α = 0.86) that were rated on a scale of 1 (*not at all true for me*) to 5 (*really true for me*). Validity was demonstrated by Elliot and Murayama (2008).

#### Analyses

Study 2 followed the same analytical plan as Study 1, with a few minor differences. During the first wave, one item from exploration in-depth was unintentionally excluded from the questionnaire (item 11; “I often reflect on which courses I like and which I don't”). As this item represents an important aspect of educational exploration in-depth that is assessed both pre- and post-transition for longitudinal research, item 11 is considered part of the complete post-transition EIPS scale. Therefore, we applied the main CFA to the second measurement wave where the item was included to examine the factor structure of the post-transition questionnaire including all EIPS items (Objective 1). The final CFA model of the second measurement wave was then also examined in the first measurement wave without item 11, to inspect the longitudinal stability of the model. The conceptual model of the post-transition scale consisted of the latent variables “reconsideration” (6 items), “exploration in-depth” (5 items), “identification with commitment” (4 items), and “self-doubt” (3 items). To test the internal and convergent validity of the EIPS (Objective 2), we used the second measurement wave for all identity subscales and all covariates except for identity style that was only assessed at W1. Finally, to test for measurement invariance over time (Objective 3), all dimensions were included in a single model as the large sample size allowed for increased model complexity^1^ The exploration in-depth item that was not assessed at W1 (i.e., item 11) was excluded from these analyses to have a comparable factor structure across time. Measurement invariance across gender was also explored using the final specified CFA model for Objective 1 in W2 data by including scale-specific adjustments. Measurement invariance was tested by constraining factor loadings to be equal between male and female adolescents to test for metric invariance and by constraining item intercepts to be equal between male and female adolescents to test for scalar invariance. Because the sample of the post-transition questionnaire was large (> 300 respondents), we applied more stringent model fit indicators as recommended by Chen (2007): Measurement invariance was assumed to hold when ΔCFI ≤ −0.010 together with RMSEA ≤ 0.015 or ΔSRMR ≤ 0.030 for metric invariance, but ΔSRMR ≤ 0.010 for scalar and full uniqueness invariance.

### Results

#### Factor Structure

**Figure 2:**
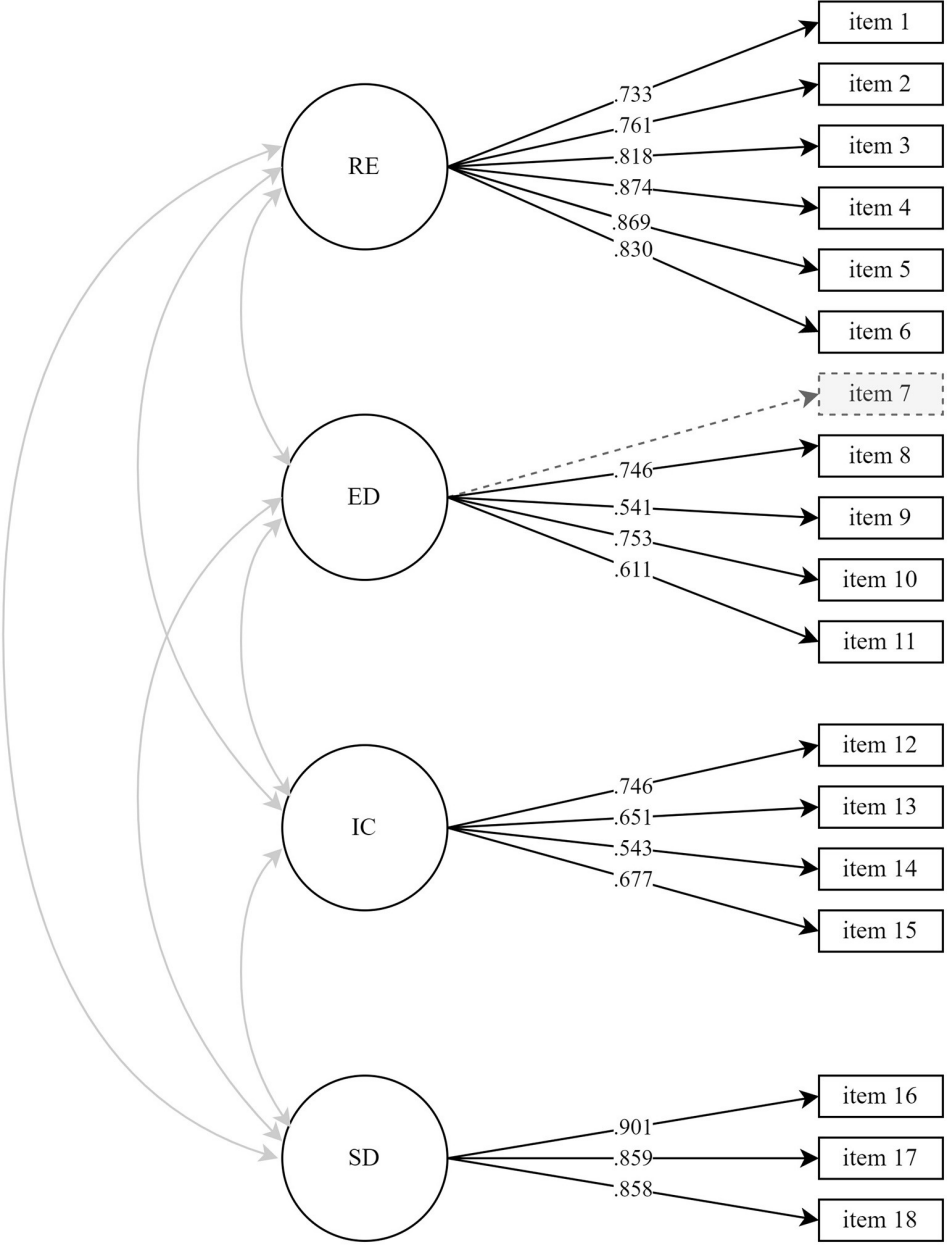
Final 4 -factor model post-transition questionnaire (W2) with standardized estimates. The dotted markings are changes from the conceptual model, indicating removal of item 4. RE, reconsideration; ED, exploration in-depth; IC, identification with commitment; SD, self-doubt. *n* = 1,191.

Findings indicated that the conceptual model did not fit the data of W2 according to two out of three fit criteria, CFI = 0.890, RMSEA = 0.080, SRMR = 0.128. After removing item 7 of exploration in-depth from the fitted model, the model showed acceptable fit according to all three criteria, CFI = 0.943, RMSEA = 0.059, SRMR = 0.062 (Figure 2 and Supplementary Table S5). The reliability of the final estimated model was overall sufficient, as indicated by α and ρ values ≥ 0.70 (Table 4 and Supplementary Table S1). Next, we examined the longitudinal stability of the model by testing the initial conceptual model in W1 and inspecting whether the CFA adjustments in W2 also improved model fit in W1. Results indicated model misfit for the conceptual model according to two out of three fit criteria, CFI = 0.890, RMSEA = 0.079, SRMR = 0.117. When applying the suggested W2 adjustment, model fit became acceptable according to all three criteria in W1, CFI = 0.936, RMSEA = 0.062, SRMR = 0.057. Overall, reliability was sufficient for the final estimated model in W1, as indicated by α and ρ values ≥ 0.70 (Supplementary Table S1). Therefore, the results generally show that after a slight adjustment, the 4-factor structure of the post-transition version of the EIPS fit well to the data.

**Table 4:** Descriptive statistics and internal validity of the post-transition EIPS at W2.

	**Descriptives**					**Zero-order correlations**		
	**α**	**M**	**SD**	**AVE**	**ASV**	**Reconsideration**	**Exploration in-depth**	**Identification with commitment**
Reconsideration	0.92	2.02	1.09	0.68	0.28	–		
Exploration in-depth	0.75	3.72	0.84	0.45	0.09	0.125^***^	–	
Identification with commitment	0.76	3.73	0.84	0.43	0.16	−0.372^***^	0.391^***^	–
Self-doubt	0.91	2.04	1.16	0.76	0.29	0.836^***^	0.062^*^	−0.426^***^

*N = 1,204. α, Cronbach's alpha of the final 4-factor model at W2. For the complete assessment of the reliability of the post-transition version of the EIPS (see Supplementary Table S1). AVE, average variance extracted; ASV, average shared variance*.

* *p < 0.05;*

**p < 0.01;

*** *p < 0.001*.

#### Validity

Providing evidence for internal validity, all correlations between the identity dimensions were in the expected directions, although effect sizes ranged from weak to strong. We observed a positive association between self-doubt and reconsideration and a negative association of identification with commitment with the dimensions of self-doubt and reconsideration (Table 4). In addition, we observed a significant positive association of exploration in-depth with reconsideration, identification with commitment, and self-doubt.

For the convergent validity of the EIPS, all hypothesized associations were in the expected directions, although effect sizes ranged from weak to strong. More optimal outcomes (i.e., more informed identity style, life satisfaction, and academic motivation, and less parental reflected doubt) were positively associated with identification with commitment, while negative outcomes (i.e., more diffused-avoidant identity style and parental reflected doubt, and less life satisfaction and academic motivation) were associated with self-doubt and reconsideration (Table 5). Additionally, we observed a positive association of normative identity style with reconsideration, identification with commitment, and self-doubt. Exploration of the convergent validity of exploration in-depth indicated a significant positive association with life satisfaction and academic motivation, both with and without controlling for self-doubt (Table 5). Further, after controlling for self-doubt, exploration in-depth was negatively associated with diffuse-avoidant identity style and parental reflected doubt. In sum, the findings of the present study generally showed support for the internal and convergent validity of the post-transition EIPS subscales.

**Table 5:** Zero-order and partial correlation testing the convergent validity of the post-transition EIPS at W2.

	**ASV**	**Informed identity style W1**	**Normative identity style W1**	**Diffused-avoidant identity style W1**	**Life satisfaction W2**	**Reflected doubt W2**	**Academic motivation W2**
**Zero-order correlations**							
Reconsideration	0.07	−0.011	0.101^***^	0.119^***^	−0.106^***^	0.593^***^	−0.058^*^
Exploration in-depth	0.06	0.389^***^	0.055	−0.052	0.103^***^	−0.053	0.465^***^
Identification with commitment	0.09	0.290^***^	0.128^***^	−0.038	0.271^***^	−0.303^***^	0.526^***^
Self-doubt	0.08	−0.060^*^	0.070^*^	0.118^***^	−0.182^***^	0.633^***^	−0.097^**^
**Partial correlations**							
Exploration in-depth	0.07	0.394^***^	0.051	−0.059^*^	0.116^***^	−0.119^***^	0.474^***^

*N = 1,204. Partial correlations are controlled for self-doubt. AVS, average shared variance*.

* *p < 0.05;*

** *p < 0.01;*

*** *p < 0.001*.

Finally, exploratory analyses further supported the validity of the post-transition version of the EIPS. Convergent validity was supported, although not all AVE values were above 0.50 (Table 4). The AVE values for exploration in-depth and identification with commitment could still be considered acceptable, since AVE is considered to be a relatively conservative indicator of convergence between items that should be considered in light of the standardized factor loadings, which are all above 0.50 (Cheung and Wang, 2017), and composite reliability of the subscales, which were all above 0.70 (Fornell and Larcker, 1981; Lam, 2012). Discriminant validity was also supported, since AVE values of each identity dimension were higher than the ASV values of other identity dimensions (Table 4) and covariates (Table 5).

#### Measurement Invariance

Findings indicated that full uniqueness invariance over time was obtained for the complete structure of the post-transition version of the EIPS, as all model fit changes were below the cutoff criteria (Table 6). Therefore, the subscales of the post-transition questionnaire could be used to examine both associations and mean-level development over time. Additionally, exploratory findings suggest similar interpretation of the post-transition version of the EIPS in male and female adolescents, as metric (ΔCFI = 0.000, ΔRMSEA = −0.001; ΔSRMR = 0.001) and scalar invariance (ΔCFI = −0.002, ΔRMSEA = 0.000; ΔSRMR = 0.001) was observed.

**Table 6:** Model fit for the different levels of post-transition measurement invariance over time.

	**CFI**	**ΔCFI**	**RMSEA**	**ΔRMSEA**	**SRMR**	**ΔSRMR**
Model 1: Configural invariance	0.945		0.043		0.053	
Model 2: Metric invariance	0.944	−0.001	0.043	0.000	0.056	0.003
Model 3: Scalar invariance	0.941	−0.003	0.043	0.000	0.057	0.001
Model 4: Full uniqueness invariance	0.940	−0.001	0.043	0.000	0.058	0.001

*All four subscales of the post-transition EIPS were included in a single model. Item 11 from exploration in-depth was excluded from analyses as it was not assessed at W1*.

## General Discussion

The present study aimed to test the psychometric properties of the pre- and post-transition versions of the EIPS questionnaire, which measures context-specific identity processes in the educational domain that are connected to the real-life challenges of adolescents. Findings indicated that after slight adjustments the factor structure of the pre- and post-transition questionnaire fit well with the data. Additionally, the identity subscales were meaningfully related to each other and measures of the self, wellbeing, parental reflected doubt, and academic motivation. Finally, in the pre-transition questionnaire, metric measurement invariance was observed for all subscales over time, and for the exploration dimensions full uniqueness invariance was additionally obtained. For the post-transition questionnaire, full uniqueness invariance across time was obtained for the complete factor structure. Therefore, the EIPS is suitable to be used to assess specific identity processes before and after the school transition through pre- and post-transition versions.

### Context Dependencies of Educational Identity Processes

The results of the present study emphasize the central role of the school transition for the meaning and relevance of educational identity processes and support the need for a questionnaire that matches the different pre- and post-transition challenges of adolescents. In a normative context where all adolescents have to choose a school, a clear distinction emerged in the adaptiveness of the mere selection of a school and feeling personally connected to the choice. Specifically, while we expected both pre-transition commitment dimensions to be associated with positive outcomes based on previous research (e.g., Luyckx et al., 2006, 2008), only identification with commitment was associated with more adaptive outcomes. Additionally, the association between exploration in-breadth and commitment making was non-significant, meaning that adolescents can select a school without broadly exploring their possibilities. When adolescents did explore their options, they generally experienced more identification with commitment and self-concept clarity. However, it should be noted that the dimension commitment making did not show a consistent fit to the data and had poor reliability, which may have influenced the results regarding the validity of this dimension and the interpretation of these results. Taken together, findings indicated that identification with a personally relevant choice is more important for optimal development than the mere making of a choice, and that exploration is related to well thought-through commitments (Marcia, 1980; Bosma, 1985).

The meaning of exploration in-breadth was found to change from the pre-transition to the post-transition phase: Whereas in the pre-transition phase, exploration in-breadth seemed an important part of optimal development, in the post-transition phase, reconsideration was no longer adaptive. More specifically, while pre-transition exploration in-breadth was strongly related to exploration in-depth and positively related to commitments, indicating the normative role of exploration in this phase, after the transition reconsideration and exploration in-depth reflected more distinct processes and reconsideration was negatively related to identification with commitment. Comparably, while pre-transition exploration in-breadth was not significantly associated with self-doubt, indicating the normative role of exploration, after the transition reconsideration had a strong positive association with self-doubt, indicating that it is not normative for adolescents to broadly explore alternatives after the transition. Additionally, adolescents who experienced higher self-doubt either pre- or post-transition also experience less adaptive outcomes, which is in line with the problematic nature of self-doubt for adolescent development (Luyckx et al., 2008; Beyers and Luyckx, 2016). Together, these findings indicate that the educational system plays a central role in determining when exploration and feelings of uncertainty may be more normative and thus less maladaptive in the development of identity, and when these experiences may suggest identity crisis among adolescents. Additionally, the dual-sided nature of identity exploration (e.g., Luyckx et al., 2008, 2013) might be more dependent on the context in which exploration behavior is enacted, as exploration in-breadth was only related to experiencing self-doubt when the context did not necessitate exploration.

Both in the pre- and post-transition context, exploration in-depth may aid in strengthening commitments (Crocetti et al., 2008; Luyckx et al., 2008), as indicated by its positive association with identity commitment and self-concept clarity. Additionally, adolescents were more motivated at school when they reflected on how well the school fits with their interests and capabilities, as indicated by the positive association for exploration in-depth with academic motivation. However, especially after the transition, adolescents seemed to be more vulnerable to perceived parental doubt about their selected school when controlling for their own degree of self-doubt. As parents are a central source for feedback when adolescents make important life choices (Koepke and Denissen, 2012), their doubt may carry more weight in the adolescents' maintenance process when the adolescents' decision is difficult to change. Therefore, within the school context the role of exploration in-depth in the strengthening of commitment and promoting academic motivation is consistent across the school transition but depending on the context, a positive or negative evaluation of the current commitments could carry more weight and thus more strongly relate to other developmental factors.

Finally, context dependencies of identity processes were also observed in terms of adolescents' interpretation of specific processes. That is, whereas full uniqueness measurement invariance was observed for all dimensions in the post-transition scale and for exploration processes in the pre-transition version, a lack of full uniqueness measurement invariance was observed in the pre-transition version for commitment processes and self-doubt. When the transition was further away, the selection of a school might be more hypothetical for adolescents compared to the moment when they were forced to apply for a specific school or already knew whether they were accepted by their preferred school. Therefore, an item such as “I know which school I want to attend” could change in meaning because this decision becomes more determined closer to the transition. As a result, the item's role within the subscale might change and influence the reliability and the measurement invariance of commitment making. Thus, in a normative context in which adolescents have been forced to choose a school regardless of their commitment, commitment measures might tap into slightly different processes than in a normative context in which adolescents have time to think about this choice. Additionally, the change in reliability and lack of measurement invariance for commitment making does not necessarily mean that commitment making cannot be assessed around a school transition, but instead, highlights the importance of critically considering the influence of the context on the identity processes. The meaning of pre-transition exploration processes does not seem to be influenced by the distance to the selection of a school. Also, after the transition the distance to the transition does not influence the adolescent's interpretation of the scale. Therefore, these findings support the central role of timing and context in studying educational identity (Bosma and Kunnen, 2001), and the need to match the assessment of identity to transitional periods (Branje et al., 2021; Christiaens et al., 2021).

### Limitations, Recommendations, and Future Directions

The findings of the present study should be considered in light of certain limitations. First, generalizability might be limited to the specific age groups and specific school contexts discussed in the present study as both age and context play an important part in identity development. That is, countries or even regions within a country may differ in the number of available options and the number of choices adolescents have. For example, how many different schools and education levels adolescents can choose from, and whether the choice is determined by grades or whether they can exert some personal preference in the choice. In the specific cross-cultural context of the present study, differences exist in the organization of the educational system between the Netherlands and Lithuania. For example, adolescents in the Netherlands have to change schools from primary to secondary education at an earlier age and generally have more options to choose from compared to adolescents in Lithuania. However, the processes adolescents engage in to develop their educational identity are expected to be similar across cultures as well as the possible impact of the transition on the relevance of these specific processes. Additionally, data were partly collected during the COVID-19 pandemic, which might have changed adolescents' behavior through for example receiving online education and limited opportunities to visit schools before the transition. Finally, as identity development can be meaningfully captured by different constellations of identity processes, future research could take a person-centered approach and study how well the EIPS captures identity statuses.

## Conclusion

The role of timing and context have been emphasized as important when studying identity (Bosma and Kunnen, 2001), particularly in the domain of educational identity which is constrained by normative educational transitions. The EIPS was developed as a context-specific identity measure to be able to take into account contextual challenges and constraints as well as context-unique elements when assessing educational identity processes. The EIPS showed good psychometric qualities for both the pre- and post-transition versions among early and middle adolescents. Additionally, findings of the present study indicated the relevance of these contextual challenges, as distance to the transition influenced the meaning of specific identity processes and the pre- and post-transition context determined whether specific processes could be considered as part of normative development. Depending on the context, some identity processes even changed in meaning from adaptive to non-adaptive for the construction of identity and for psychosocial functioning. Therefore, this study provides evidence that the EIPS can be used in future research to capture the central role of the school transition in identity processes and to facilitate context-specific identity research in the educational domain.

## Data Availability Statement

The datasets presented in this study can be found in online repositories. The names of the repository/repositories and accession number(s) can be found at: https://osf.io/mscx9/.

## Ethics Statement

The studies involving human participants were reviewed and approved by the Local Ethics Board of Utrecht University (protocol nos. FETC18-135, FETC20-157) and by the Civil Society and Sustainability Research Group at the Faculty of Social Sciences, Arts, and Humanities, Kaunas University of Technology (protocol no. V19-1253-12). Written informed consent to participate in this study was provided by the participants' legal guardian/next of kin.

## Author Contributions

AC wrote the first draft of the manuscript. SN and SB were most intensively involved with manuscript revision. AC and RV were responsible for organizing the database of Study 1 and Study 2, respectively. AC performed the statistical analyses that were supervised by SN. EM contributed to the theoretical framework and background of the development of the EIPS. SB and RE conceived and acquired funding for the projects that were used in Study 1 and 2, respectively. All authors contributed to the conception and design of the study. All authors contributed to manuscript revision, read, and approved the submitted version.

## Funding

This study has received funding from European Regional Development Fund (Project No. 01.2.2-LMT-K-718-03-0059) under grant agreement with the Research Council of Lithuania (LMTLT) and from a grant of the European Research Council (ERC-2017-CoG - 773023 INTRANSITION).

## Conflict of Interest

The authors declare that the research was conducted in the absence of any commercial or financial relationships that could be construed as a potential conflict of interest.

## Publisher's Note

All claims expressed in this article are solely those of the authors and do not necessarily represent those of their affiliated organizations, or those of the publisher, the editors and the reviewers. Any product that may be evaluated in this article, or claim that may be made by its manufacturer, is not guaranteed or endorsed by the publisher.

## Footnotes

^1^Testing measurement invariance separately for each dimension, findings remained the same when we relaxed the equality constraints of the item intercept of item 15 of identification with commitment and item 8 of exploration in-depth.
