# Supplementary material for: Psychometric Qualities of the Educational Identity Processes Scale (EIPS)
Source: Front Psychol. 2022 Apr 19;13:861220. doi: 10.3389/fpsyg.2022.861220 (PMC9063563; doi:10.3389/fpsyg.2022.861220)
Supplement: Supplementary file 1 [file Data_Sheet_1.docx]

Supplementary Material

**Table S1.** *Reliability statistics for the EIPS subscales.*

|  | Pre-transition EIPS | | | | | | | | Post-transition EIPS | | | | | | | |
| --- | --- | --- | --- | --- | --- | --- | --- | --- | --- | --- | --- | --- | --- | --- | --- | --- |
|  | Wave 1 (*n* = 230) | | | | Wave 2 (*n* = 191) | | | | Wave 1 (*n* = 1,258)^a^ | | | | Wave 2 (*n* = 1,191) | | | |
|  | Conceptual model | | Final 5-factor model | | Conceptual model | | Final 5-factor model | | Conceptual model | | Final 4-factor model | | Conceptual model | | Final 4-factor model | |
|  | α | ρ | α | ρ | α | ρ | α | ρ | α | ρ | α | ρ | α | ρ | α | ρ |
| Exploration in-breadth/Reconsideration | .88 | .88 | .88 | .88 | .86 | .86 | .86 | .86 | .90 | .90 | .90 | .90 | .92 | .93 | .92 | .93 |
| Exploration in-depth | .86 | .86 | .84 | .80 | .84 | .84 | .82 | .80 | .61 | .67 | .71 | .72 | .69 | .73 | .75 | .76 |
| Commitment making | .70 | .71 | .70 | .64 | .49 | .54 | .49 | .53 |  |  |  |  |  |  |  |  |
| Identification with commitment | .84 | .84 | .84 | .84 | .75 | .75 | .75 | .75 | .74 | .75 | .74 | .75 | .76 | .75 | .76 | .75 |
| Self-doubt | .82 | .84 | .83 | .83 | .87 | .88 | .88 | .88 | .88 | .88 | .88 | .88 | .91 | .91 | .91 | .91 |

Note. Cutoff criterion for good reliability was indicated by a value ≥ .70. α = Cronbach’s alpha (Cronbach, 1951), ρ = Raykov’s rho (i.e., composite reliability; Raykov, 2001). ^a^ item 11 was unintentionally excluded.

**Table S2.** *Pre-transition questionnaire set-up of the Educational Identity Processes scale.*

| *EIPS* | *Adapted from* |
| --- | --- |
| *Exploration in-breadth* |  |
| 1. I often think about which school would fit me best. | I try to regularly assess which education ultimately is best for me. (UMICS, item 16) |
| 1. I think actively about which school I like most. | Thinking about how I could fit into many different careers. (VISA, item 3) |
| 1. I am comparing different schools to find out which school fits me best. | I am considering a number of different lifestyles that might suit me. (DIDS, item 8). |
| 1. I often talk with other people about which schools I can attend. | I often talk with other people about my education. (UMICS, item 10 ED) |
| 1. I try to find out a lot about the different schools I can attend. | Learning about various jobs that I might like. (VISA, item 4 EB) |
| *Exploration in-depth* |  |
| 1. I often talk with other people about whether the school I want to attend fits me. | I try to find out what other people think about the specific direction I decided to take  in my life. (DIDS, item 24) |
| 1. I try to find out a lot about what I am good at and less good at in my school. | Learning as much as I can about the particular educational requirements of the career that interests me the most. (VISA, item 2 ED) |
| 1. I often talk with other people about the things I like about the school I want to attend. | I talk with other people about my plans for the future. (DIDS, item 22) |
| 1. I often reflect on which courses I am good at and less good at. | Identifying my strongest talents as I think about careers. (VISA, item 1 ED) |
| 1. I often reflect on which courses I like and which I don’t. | Thinking about all the aspects of working that are important to me. (VISA, item 5 ED) |
| *Commitment making* |  |
| 1. I know which school I want to attend. | I know what kind of work is best for me. (VISA, item 1 CO) |
| 1. The school I have chosen seems much nicer to me than other schools. | No other career is as appealing to me as the one I expect to enter. (VISA, item 2 CO) |
| 1. No one can change my mind about which education I want to attend. | No one will change my mind about the career I have chosen. (VISA, item 4 CO) |
| *EIPS* | *Adapted from* |
| *Identification with commitment* |  |
| 1. The school I want to attend really fits me. | I sense that the direction I want to take in my life will really suit me. (DIDS, item 19) |
| 1. The school I want to attend allows me to do the things I want in the future. | Becoming a worker in my chosen career will allow me to become the person I dream to be. (VISA, item 3 IC) |
| 1. I expect to learn things I find interesting at the school I want to attend. | My plans for the future match with my true interests and values. (DIDS, item 16) |
| 1. I am willing to put in a lot of effort to be able to go to the school I want to attend. | I have invested a lot of energy into preparing for my chosen career. (VISA, item 5 CO) |
| - *Self-Doubt* |  |
| 1. I am doubtful about which school would fit me best. | I am doubtful about what I really want to achieve in life. (DIDS, item 11) |
| 1. Thinking about which school I want to attend, makes me feel uneasy. | Thinking about choosing a career makes me feel uneasy. (VISA, item 1 SD) |
| 1. I doubt I will find a school that really fits me. | I doubt I will find a career that suits me. (VISA, item 4 SD) |
| 1. I keep wondering if I can attend the school which I like to attend best. | I may not be able to get the job I really want. (VISA, item 5 SD) |
| 1. I worry about which school I really like. | I worry about what I want to do with my future. (DIDS, item 12) |

**Table S3.** *CFA model adjustments of the pre-transition EIPS questionnaire.*

|  | ΔCFI | ΔRMSEA | ΔSRMR | Suggestion for improvement | Conceptual reasoning |
| --- | --- | --- | --- | --- | --- |
| Model 1 | .829 | .087 | .085 | Suggested cross-loading for item 6 from exploration in-depth on exploration in-breadth | The item captures exploration behavior and has overlap with item 4 from exploration in-breadth as both items capture “I often talk with other people about…” and only differ in the content of what is discussed. However, as cross-loading were not allowed, item 6 will be removed from the scale. |
| Model 2 | .023 | -.006 | -.003 | Suggested cross-loading for item 18 from self-doubt on commitment making | The item captures doubt about “which school would fit me best”, which is in contrast to commitment making which discusses items such as “I know which school I want to attend.” However, as cross-loading is not allowed, item 18 will be removed from the scale. |
| Model 3 | .011 | -.001 | -.002 | No further suggestions at the item level, but an indication for correlation of error terms of items 9 and 10 from exploration in-depth | The items are from the same subscale and both discuss the reflection on courses (“I often reflect on which courses…”), but differ in the content of what information is used for reflection (i.e., capability or liking). Still, items are similar enough to have shared residual variance. |
|  | ΔCFI | ΔRMSEA | ΔSRMR | Suggestion for improvement | Conceptual reasoning |
| Model 4 | .018 | -.006 | -.005 | Suggested correlation of error terms for items 11 and 12 from commitment making | The items are from the same subscale and discuss the degree to which they are certain about which school they want to attend more lightly as opposed to their resistance to being swayed (item 13). Therefore, these items are similar enough to have shared residual variance. |
| Model 5 | .007 | -.002 | -.001 | Suggestion for correlated error terms for item 4 from exploration in-breadth and item 8 from exploration in-depth | Although the items belong to different subscales, both items were rooted in exploration behavior by discussing their educational identity with other people (“I often talk with other people about…”). Therefore, these items are similar enough to have shared residual variance. |
| Model 6 | .009 | -.002 | -.001 | Suggestion for correlated error terms for item 11 from commitment making and item 22 from self-doubt. | Although the items belong to different subscales, item 22 discusses the doubt about which school the adolescent likes, while item 11 discusses whether the adolescent knows which school they want to attend. The direct contrast between the items captures the negative association between self-doubt and commitment making and possibly explains the shared residual variance. |
|  | ΔCFI | ΔRMSEA | ΔSRMR | Suggestion for improvement | Conceptual reasoning |
| Model 7 | .010 | -.004 | -.001 | Acceptable model fit was reached according to all three indicators |  |

*Note.*  Model 1 tested the conceptual model. Model 2 tested model 1 without item 6. Model 3 tested model 2 without item 18. Model 4 tested model 3 with the addition of a correlation between the error terms of items 9 and 10. Model 5 tested model 4 with the addition of correlated error terms between items 11 and 12. Model 6 tested model 5 with the addition of correlated error terms between items 4 and 8. Model 7 tested model 6 with the addition of correlated error terms between items 11 and 22.

**Table S4.** *Post-transition questionnaire set-up of the Educational Identity Processes scale.*

| *EIPS* | *Adapted from* |
| --- | --- |
| *Reconsideration* |  |
| 1. I am comparing different schools to find out if another school would fit me better. | I try to regularly assess which education ultimately is best for me. (UMICS, item 16) |
| 1. I often talk with other people about which other schools I can attend. | I often talk with other people about my education. (UMICS, item 10) |
| 1. I try to find out a lot about the other schools I can attend. | Learning about various jobs that I might like. (VISA, item 4 EB) |
| 1. I often think about looking for a different school. | I often think it would be better to try to find a different education. (UMICS, item 11) |
| 1. I often think that a different school would fit me better. | I often think that a different education would make my life more interesting. (UMICS, item 12) |
| 1. Actually, I am looking for a different school. | In fact, I’m looking for a different education. (UMICS, item 13) |
| *Exploration in-depth* |  |
| 1. I often talk with other people about whether my school fits me. | I try to find out what other people think about the specific direction I decided to take in my life. (DIDS, item 24) |
| 1. I try to find out a lot about what I am good at and less good at in my school. | Learning as much as I can about the particular educational requirements of the career that interests me the most. (VISA, item 2 ED) |
| 1. I often talk with other people about the things I like about my school. | I talk with other people about my plans for the future. (DIDS, item 22) |
| 1. I often reflect on which courses I am good at and less good at. | Identifying my strongest talents as I think about careers. (VISA, item 1 ED) |
| 1. I often reflect on which courses I like and which I don't. | Thinking about all the aspects of working that are important to me. (VISA, item 5 ED) |
| *EIPS* | *Adapted from* |
| *Identification with commitment* |  |
| 1. My school really fits me. | I sense that the direction I want to take in my life will really suit me. (DIDS, item 19) |
| 1. My school allows me to do the things I want in the future. | Becoming a worker in my chosen career will allow me to become the person I dream to be. (VISA, item 3 IC) |
| 1. In my school I learn things I find interesting. | My plans for the future match with my true interests and values. (DIDS, item 16) |
| 1. I am willing to put in a lot of effort to stay at the school of my choice. | I have invested a lot of energy into preparing for my chosen career. (VISA, item 5 CO) |
| - *Self-Doubt* |  |
| 1. I am doubtful about whether my school really fits me. | I doubt I will find a career that suits me. (VISA, item 4 SD) |
| 1. Thinking about whether my school fits me, makes me feel uneasy. | Thinking about choosing a career makes me feel uneasy. (VISA, item 1 SD) |
| 1. I worry if I really like my school. | I worry about what I want to do with my future. (DIDS, item 12) |

**Table S5.** *CFA model adjustments of the post-transition EIPS questionnaire.*

|  | ΔCFI | ΔRMSEA | ΔSRMR | Suggestion for improvement | Conceptual reasoning |
| --- | --- | --- | --- | --- | --- |
| Model 1 | .890 | .080 | .128 | Suggested cross-loading for item 7 from exploration in-depth on exploration in-breadth. | The item captures exploration behavior and has overlap with item 2 from exploration in-breadth as both items capture “I often talk with other people about…” and only differ in the content of what is discussed. However, as cross-loading were not allowed, item 7 will be removed from the scale. |
| Model 2 | .053 | -.021 | -.066 | Acceptable model fit was reached according to all three indicators |  |

*Note.* W2 was used for analyses. *n* = 1191.

**Table S6.** *Overview of the Parental Reflected Doubt scale.*

| 1. My parents doubt whether the education I want to attend really fits me. |
| --- |
| 1. My parents doubt whether I can attain the level of schooling of my education. |
| 1. My parents do not expect me to get far in my education. |
| 1. My parents have little confidence in my scholastic abilities. |
| 1. My parents would want me to get higher grades. |
| 1. My parents would prefer if I would follow a different education.^a^ |

Note. The items were slightly adjusted to match the pre- and post-transition experiences of the adolescent.

^a^ Item 6 was only assessed post-transitions, since the primary school adolescents attend is decided by the parents and is thus not informative to assess in the pre-transition early adolescent sample.
